# Attitudes toward uncertain results from prenatal exome sequencing: a national survey among healthcare professionals working in the prenatal setting

**DOI:** 10.3389/fmed.2024.1335649

**Published:** 2024-05-15

**Authors:** Dongfang Lu, Jing Yang, Wei Shen, Min Chen

**Affiliations:** ^1^Department of Obstetrics, Affiliated Xiaoshan Hospital, Hangzhou Normal University, Hangzhou, China; ^2^Department of Obstetrics and Gynecology, Department of Fetal Medicine and Prenatal Diagnosis, Key Laboratory for Major Obstetric Diseases of Guangdong Province, The Third Affiliated Hospital of Guangzhou Medical University, Guangzhou, China

**Keywords:** uncertain results, prenatal exome sequencing, attitudes, survey, genetic counseling

## Abstract

**Objective:**

The objective of this study was to investigate the attitudes of healthcare professionals (HPs) working in the prenatal setting toward uncertain results (UR) from prenatal exome sequencing (pES) in China.

**Methods:**

We conducted a national survey among HPs working in the prenatal setting. UR in our study include variants of uncertain significance (VUS), variants with variable penetrance/expressivity (VVPE), and secondary findings unrelated to the indication for testing (SFs). A total of 285 questionnaires that met the inclusion criteria were collected. Data were analyzed using IBM SPSS Statistics 26.

**Results:**

When performing the pre-test counseling, only 7.4% of HPs mentioned the possibility of VUS, 6.3% discussed the possibility of VVPE, and 7.4% introduced the SFs with parents with the option to not report these variants. In post-test counseling, 73.0–82.8% HPs discussed with the parents but did not make any recommendations for managing the pregnancy after reporting UR (73.0% for VUS, 82.8% for VVPE, 74.7% for SFs, respectively).

**Conclusion:**

Most parents did not have the option of opting out of reporting UR from pES in pre-test counseling. UR did not influence the pregnancy recommendation made by most HPs. Establishing national guidelines for reporting UR from pES and developing strategies to improve counseling skills may help HPs manage UR.

## 1 Introduction

Aneuploidy and copy number variation are present in about 40% of fetuses with a congenital anomaly, which can be detected using classical G-banding karyotype and chromosome microarray analysis (1). Next generation sequencing, including both exome and genome sequencing, has revolutionized the field of clinical genetics (2). The exome comprises 1–2% of the human genes but contains approximately 85% of known disease-related variants (3, 4). In the presence of congenital anomalies, prenatal exome sequencing (pES) provides an additional diagnostic yield of 8.5–10.3% compared to conventional genetic testing in unselected fetuses (5, 6). This number grows to between 15.4% and 18.9% in fetuses with multiple anomalies (5, 6).

While pES has increased the number of genetic diagnoses in pregnancy, there are practical and ethical challenges in interpreting the results for parents (7). In addition, pES has a greater potential to cause uncertain results (UR) (8). UR may emerge for several reasons. For example, there may be a variant of uncertain significance (VUS) being identified through pES but whose significance to the health of a baby is not known (9). Different people with the same genetic condition may have variable expressivity or incomplete penetrance (10, 11). However, UR from pES may affect clinical decision-making or have adverse psychological effects on parents. The uncertainty surrounding the outcome of the baby’s prognosis, as well as the general lack of information about the future, made it difficult for parents to make decisions (12). In addition, parents struggled to cope with having to make decisions in such a short period of time (13). The emotional impact of UR can create feelings of worry, fear and ongoing anxiety (14, 15). A number of studies looking at parents’ attitudes toward uncertainty from pES have been published in the last decade (8, 16–20). There were also some studies about the views of healthcare professionals (HPs) on pES (21–24).

Concerns regarding the need for appropriate consent, the complexity of genomic data, the ongoing need for reanalysis and recontact, and the challenges of interpreting results in a meaningful way for patients have been raised (7, 21).

Recent ACMG guidelines on the use of pES advocate that laboratories should have clear policies for what types of variants, including VUS, will be reported and recommends that pre-test counseling includes discussion of the potential to identify VUS as well as adult-onset diseases in the fetus (25). Royal College of Obstetricians and Gynaecologist (RCOG) proposed that detailed pre-test and post-test parental counseling must be provided by professionals experienced in sequencing to explain the potential technical and ethical limitations, and to discuss options on which results will be available, including VUS and secondary findings (1). International Society for Prenatal Diagnosis (ISPD) stated that the approach to reporting VUS should be disclosed during pre-test counseling and included in consenting (26). These three professional bodies proposed the same thing: that the potential for VUS should be discussed during pre-test counseling.

In China, there is no national consensus on reporting UR from pES. Practices vary in different prenatal settings in China. A local consensus recommended that the report of VUS should be based on clinical conditions (27). If the variants to be reported involve variable expressivity or incomplete penetrance, it should be indicated and it was suggested to indicate penetrance data and annotate references (27). For secondary findings unrelated to the indication for testing, whether to be reported should be discussed in the pre-test counseling (27).

Currently, there are few studies on HPs’ attitudes toward UR from pES in China. The lack of research in this area is an obstacle to establishing national guidelines for reporting UR from pES and developing strategies to improve counseling skills to help HPs manage UR. An insight into the attitudes of HPs will provide us with new insights and a better understanding in this respect. Our study aimed to investigate the different attitudes of HPs working in the prenatal setting toward UR from pES and describe how these UR are managed in China.

## 2 Materials and methods

### 2.1 Study design

The survey was designed based on a literature review (8, 16–24) and examined for content validity by three specialists in prenatal diagnosis outside the study team. Several rigor criteria were considered to evaluate the scientific quality of the research, such as credibility, transferability and dependability. A pilot study was conducted to test the clarity and layout of the questions by 20 HPs working in the prenatal settings who had experience with pES. Minor revisions were made to the language to improve the readability of the content. It was a national online survey among HPs working in the prenatal setting, including specialists in prenatal diagnosis and fetal medicine, obstetricians, technicians in prenatal diagnosis laboratories, clinical geneticists, laboratory geneticists, and genetic counselors. UR from pES in our study include variants of uncertain significance (VUS), variants with variable penetrance/expressivity (VVPE), and secondary findings unrelated to the indication for testing (SFs). The questionnaire commenced with a cover page describing the aims of the study, consent, and confidentiality. Participation was voluntary. The informed consent on the cover page has been reviewed by the Institutional Review Board (IRB) to assure human subject protection and research integrity. A survey instrument^1^ was used for data collection. Identification (ID) numbers unique to the entire study were assigned when the questionnaires were submitted. The questionnaire comprised five sections: (1) demographic data; (2) practice; (3) attitudes; (4) recommendations after reporting UR; and (5) views of the reclassification of the VUS. For details of the questionnaire, please see the Supplementary Figure S1.

### 2.2 Ethical approval

Approval was granted by the Ethics Committees of Affiliated Xiaoshan Hospital, Hangzhou Normal University (25th January, 2022/no. KL2022011). All participants gave their informed consent in this study.

### 2.3 Recruitment of participants

Inclusion criteria were HPs working in the prenatal settings who had experience with pES in China. At the beginning of the questionnaire, we asked participants if they had any experience with pES. If the participant selects “No,” the questionnaire will be excluded from further analysis.

The study was conducted with a convenient sample of HPs working in prenatal settings throughout China. Survey respondents were recruited in one of two ways.

1. QR code. Participants were recruited through an electronic poster containing information about the survey and a WeChat (Tencent) QR code linked to the online questionnaire. We sent this electronic poster to different WeChat groups containing HPs and shared it on WeChat moments. At the same time, we asked HPs to help forward this electronic poster. The participants completed the questionnaires using their mobile phones after scanning the QR code for the study.
2. E-mail. We sent e-mails to HPs working in prenatal settings in China whose e-mail addresses were in our address book. The participants completed the questionnaires by clicking on the URL link provided in their e-mail.

The WeChat and E-mail online survey allowed investigator access to demographically diverse respondents from across the whole country. The geographical distribution of respondents is shown on a map (Supplementary Figure S2). A total of 295 questionnaires were collected from 21 February 2022 to 21 June 2023, among which 294 participants completed the survey through QR code (294/294, a 100% response rate; this only relates to those who completed the survey when approached, as we could not identify how many people viewed the survey invitation and we are unable to detect how many people started but withdrew from the survey later). Only one participant completed the survey through E-mail (1/20, a 5% response rate). Ten questionnaires were excluded from further analysis because they had no experience with pES, leaving 285 for the final analysis.

### 2.4 Data analysis

Statistical analyses were performed using IBM SPSS Statistics 26.

(SPSS, Inc., Chicago, IL, United States). Descriptive statistics were generated as frequencies and percentages for categorical data. Differences were assessed using the chi-square test, with *p* < 0.05 considered significant.

## 3 Results

### 3.1 Demographic data

The demographic characteristics of participants including age, gender, educational level, years in practice, level of experience, ethnicity, religious beliefs, and practice setting are summarised in Table 1. In the whole cohort, 258 (90.5%) were female and 27 (9.5%) were male. The mean age was 43.2 ± 8.4 years (range, 24–66 years). The 285 participants comprised 68 (23.9%) specialists in prenatal diagnosis and fetal medicine, 161 (56.5%) obstetricians, 20 (7.0%) technicians in prenatal diagnosis laboratories, 17 (6.0%) clinical geneticists, 13 (4.6%) laboratory geneticists, 6 (2.1%) genetic counselors. The level of education reported by respondents included 23 (8.1%) doctors, 79 (27.7%) masters, 167 (58.6%) bachelors, and 16 (5.6%) college degrees and below. Most participants worked in general hospitals (56.5%) and maternal and neonatal hospitals (29.1%).

**Table 1:** Demographic data.

Characteristic		*N* (%)
Specialty	Specialist in prenatal diagnosis and fetal medicine	68 (23.9)
	Technicians in prenatal diagnosis laboratories	20 (7.0)
	Clinical geneticists	17 (6.0)
	Obstetricians	161 (56.5)
	Laboratory geneticists	13 (4.6)
	Genetic counselors	6 (2.1)
Years in practice	0–5	23 (8.1)
	6–10	51 (17.9)
	11–20	81 (28.4)
	>20	130 (45.6)
Level of experience	Junior	29 (10.2)
	Intermediate	75 (26.3)
	Senior	181 (63.5)
Age, mean (SD), y		43.2 ± 8.4
Gender	Male	27 (9.5)
	Female	258 (90.5)
Ethnicity	Han	246 (86.3)
	Ethnic minorities	39 (13.7)
Religion	None	256 (89.8)
	Christian	2 (0.7)
	Buddhism	16 (5.6)
	Muslim	7 (2.5)
	Others	4 (1.4)
Educational level	Doctor	23 (8.1)
	Master	79 (27.7)
	Bachelor	167 (58.6)
	College degree and below	16 (5.6)
Practice setting	General hospital	161 (56.5)
	Maternal and neonatal hospital	83 (29.1)
	Genetics hospital	2 (0.7)
	Private practitioner	14 (4.9)
	Urban or rural primary healthcare center	2 (0.7)
	Genomics institution	18 (6.3)
	Others	5 (1.8)

### 3.2 Practices

#### 3.2.1 Who decides UR to report?

**Figure 1:**
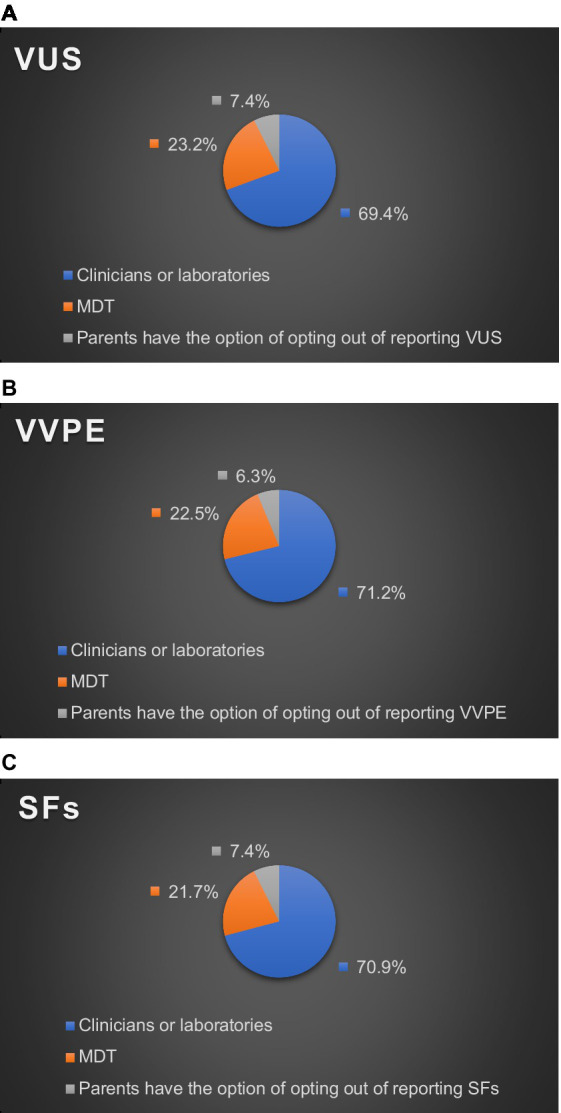
Who decides uncertain results to report? **(A)** VUS. **(B)** VVPE. **(C)** SFs.

Reporting practices for UR varied in different practice settings. As for who made the final decision on returning VUS in their practice settings, 69.4% of HPs chose clinicians or laboratories, and 23.2% chose Multi-Disciplinary Team (MDT). MDT members include specialists in prenatal diagnosis and fetal medicine, geneticists, technicians in prenatal diagnosis laboratories, and obstetricians involved in prenatal diagnosis. Only 7.4% of HPs reported that their practice settings gave parents the option to opt out of VUS (Figure 1A).

As for who made the final decision on returning VVPE in their practice settings, 71.2% of HPs chose clinicians or laboratories, and 22.5% chose MDT. Only 6.3% of HPs reported that their practice settings gave parents the option to opt out of VVPE (Figure 1B).

As for who made the final decision on returning SFs in their practice settings, 70.9% of HPs chose clinicians or laboratories, and 21.7% chose MDT. Only 7.4% of HPs reported that their practice settings gave parents the option to opt out of SFs (Figure 1C).

#### 3.2.2 Who provides pre-test counseling or post-test counseling?

**Figure 2:**
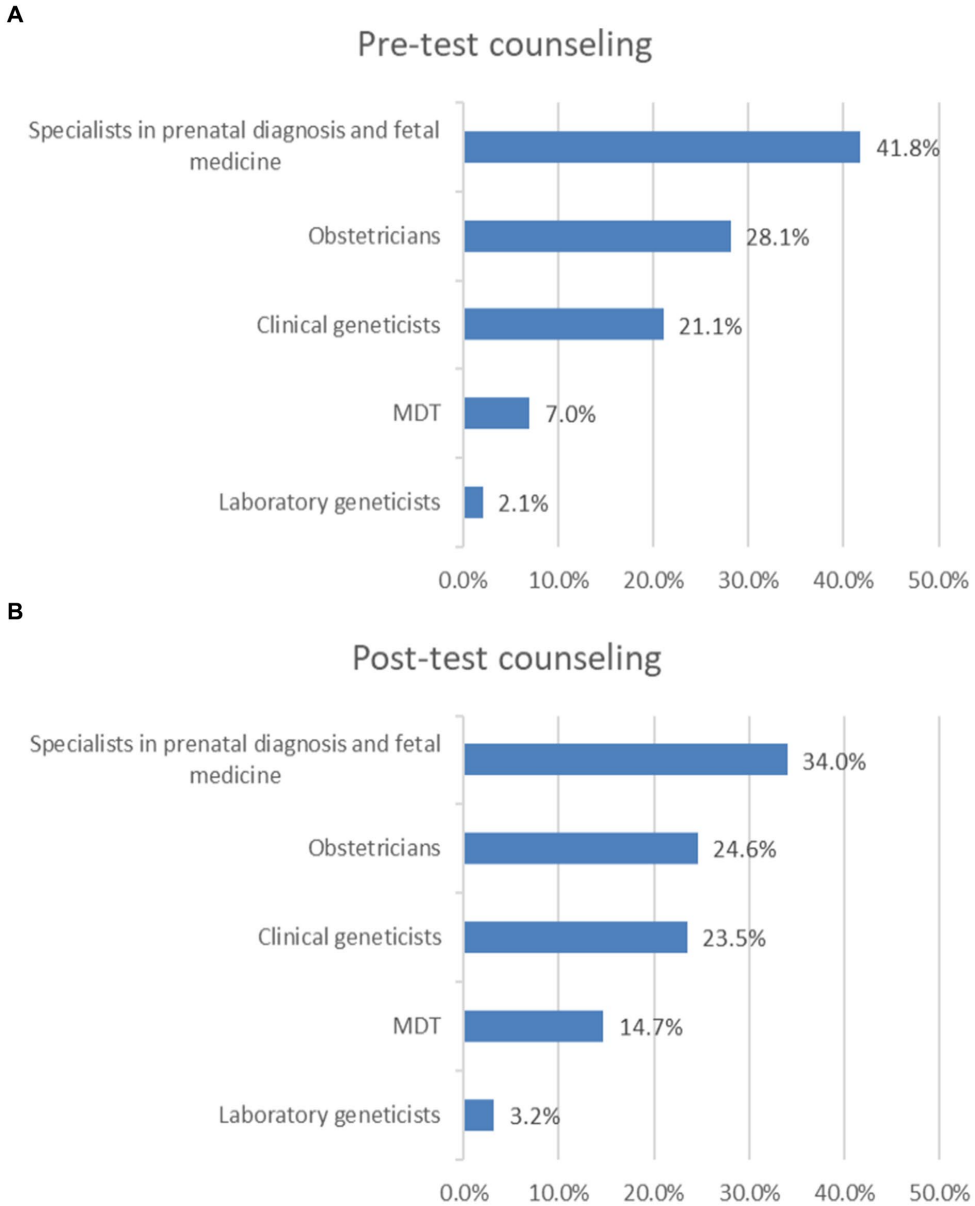
Who provides pre-test counseling or post-test counseling? **(A)** Pre-test counseling. **(B)** Post-test counseling.

In pre-test counseling, 41.8% of survey respondents reported that pre-test counseling is provided by specialists in prenatal diagnosis and fetal medicine in their practice settings, 28.1% chose obstetricians, 21.1% chose clinical geneticists, 7.0% chose MDT, and 2.1% chose laboratory geneticists (Figure 2A).

In post-test counseling, 34.0% of survey respondents reported that post-test counseling is provided by specialists in prenatal diagnosis and fetal medicine in their practice settings, 24.6% chose obstetricians, 23.5% chose clinical geneticists, 14.7% chose MDT, 3.2% chose laboratory geneticists (Figure 2B).

### 3.3 Attitudes

#### 3.3.1 Attitudes toward the return of UR from pES

Regarding the return of UR from pES, 57.2–64.9% of HPs agreed that UR should be returned to pregnant individuals (57.2% for VUS, 64.9% for VVPE, 62.5% for SFs, respectively), 1.8–3.5% of HPs thought that UR should not be returned to pregnant individuals (3.5% for VUS, 2.1% for VVPE, 1.8% for SFs, respectively), and 33.0–39.3% of HPs felt that the question of whether to return the UR should be discussed with the pregnant individual during pre-test counseling (39.3% for VUS, 33.0% for VVPE, 35.8% for SFs, respectively) (Table 2).

**Table 2:** Attitudes toward the return of UR from pES.

Opinions	*N* (%)		
	VUS	VVPE	SFs
UR should be returned to pregnant individuals	163 (57.2)	185 (64.9)	178 (62.5)
UR should not be returned to pregnant individuals	10 (3.5)	6 (2.1)	5 (1.8)
Whether to return the UR should be discussed with the pregnant individual during pre-test counseling.	112 (39.3)	94 (33.0)	102 (35.8)

UR, uncertain results; pES, prenatal exome sequencing; VUS, variants of uncertain significance; VVPE, variants with variable penetrance/expressivity; SFs, secondary findings unrelated to the indication for testing.

Differences in specialty, years in practice, gender and educational level caused some differences in participants’ attitudes toward the return of UR (*p* < 0.05) (Table 3).

**Table 3:** Differences in attitudes toward the return of UR among participants with different demographic characteristics.

Demographics	VUS		VVPE		SFs	
	c2	P	c2	P	c2	P
Specialty	29.936	0.001	18.294	0.05	18.596	0.046
Years in practice	13.678	0.033	8.134	0.228	11.726	0.068
Level of experience	4.574	0.334	4.491	0.344	4.817	0.307
Age	83.937	0.122	68.403	0.532	71.929	0.414
Gender	2.047	0.359	6.462	0.04	6.174	0.046
Ethnicity	0.148	0.929	1.148	0.563	2.15	0.341
Religion	3.059	0.931	3.541	0.896	3.625	0.889
Educational level	19.172	0.004	8.712	0.19	8.025	0.236

#### 3.3.2 Whether UR from pES affect the doctor-patient relationship

When UR from pES are returned to parents who were not aware of UR before testing, 26.0% of HPs agreed that the UR would affect the doctor-patient relationship, 8.4% disagreed, and 65.6% thought it depended on the parent’s attitude towards the UR.

Although more than 50% of HP believe that whether it affects the doctor-patient relationship depends on the parent’s attitude towards the UR, clinical geneticists are not the same as HP in other specialties. More than 50% of clinical geneticists believe that UR affects the doctor-patient relationship (Table 4).

**Table 4:** Differences in attitudes toward the question of whether UR affects the doctor-patient relationship among participants with different specialty.

Specialty		Yes	No	Depend	Total
Specialist in prenatal diagnosis and fetal medicine	N	16	7	45	68
	%	23.5%	10.3%	66.2%	100.0%
Technicians in prenatal diagnosis laboratories	N	3	1	16	20
	%	15.0%	5.0%	80.0%	100.0%
Clinical geneticists	N	9	1	7	17
	%	52.9%	5.9%	41.2%	100.0%
Obstetricians	N	43	15	103	161
	%	26.7%	9.3%	64.0%	100.0%
Laboratory geneticists	N	2	0	11	13
	%	15.4%	0.0%	84.6%	100.0%
Genetic counselors	N	1	0	5	6
	%	16.7%	0.0%	83.3%	100.0%
Total	N	74	24	187	285
	%	26.0%	8.4%	65.6%	100.0%

### 3.4 Recommendations after reporting UR

#### 3.4.1 Recommendations for continuing or terminating a pregnancy after reporting UR

In post-test counseling, when UR are returned to parents, 11.2–22.1% of HPs recommended continuing the pregnancy after discussing with the parents (22.1% for VUS, 11.2% for VVPE, 15.4% for SFs, respectively), 4.9–9.8% recommended termination of the pregnancy after discussing with the parents (4.9% for VUS, 6.0% for VVPE, 9.8% for SFs, respectively), and 73.0–82.8% discussed with the parents but did not make any recommendations (73.0% for VUS, 82.8% for VVPE, 74.7% for SFs, respectively) (Table 5).

**Table 5:** Recommendations for continuing or terminating a pregnancy after reporting UR.

Recommendations	*N* (%)		
	VUS	VVPE	SFs
Continuing the pregnancy	63 (22.1)	32 (11.2)	44 (15.4)
Terminating the pregnancy	14 (4.9)	17 (6.0)	28 (9.8)
Discussed with the parents but did not make any recommendations	208 (73.0)	236 (82.8)	213 (74.7)

UR, uncertain results; VUS, variants of uncertain significance; VVPE, variants with variable penetrance/expressivity; SFs, secondary findings unrelated to the indication for testing.

#### 3.4.2 Recommendations to the parents who felt overwhelmed by the UR from pES

When parents felt overwhelmed by the UR from pES, 16.1% of HPs provided their options of termination or continuation of the pregnancy, 12.6% made no recommendation, 6.7% found themselves in a dilemma and could do nothing to help parents, 16.1% signposted parents to psychological support, 28.1% made a referral, and 20.4% requested consultation by senior staff (Table 6).

**Table 6:** Recommendations to the parents who felt overwhelmed by UR from pES.

Recommendations	*N* (%)
Referral	80 (28.1)
Requested consultation by senior staff	58 (20.4)
Provided their own options of termination or continuation of the pregnancy	46 (16.1)
Signposted parents to psychological support	46 (16.1)
No recommendation	36 (12.6)
Found themselves in a dilemma and could do nothing to help patients	19 (6.7)

UR, uncertain results; pES, prenatal exome sequencing.

### 3.5 Views of the reclassification of the VUS

#### 3.5.1 Views of the reanalysis and recontacting the patients when VUSs were reclassified years after the original test

In our study, 64.9% of HPs would reanalyse the results when VUSs were reclassified years after the original test. However, only 21.4% of HPs would recontact the patients.

#### 3.5.2 Responsibility of laboratories and clinicians with regard to VUSs

**Figure 3:**
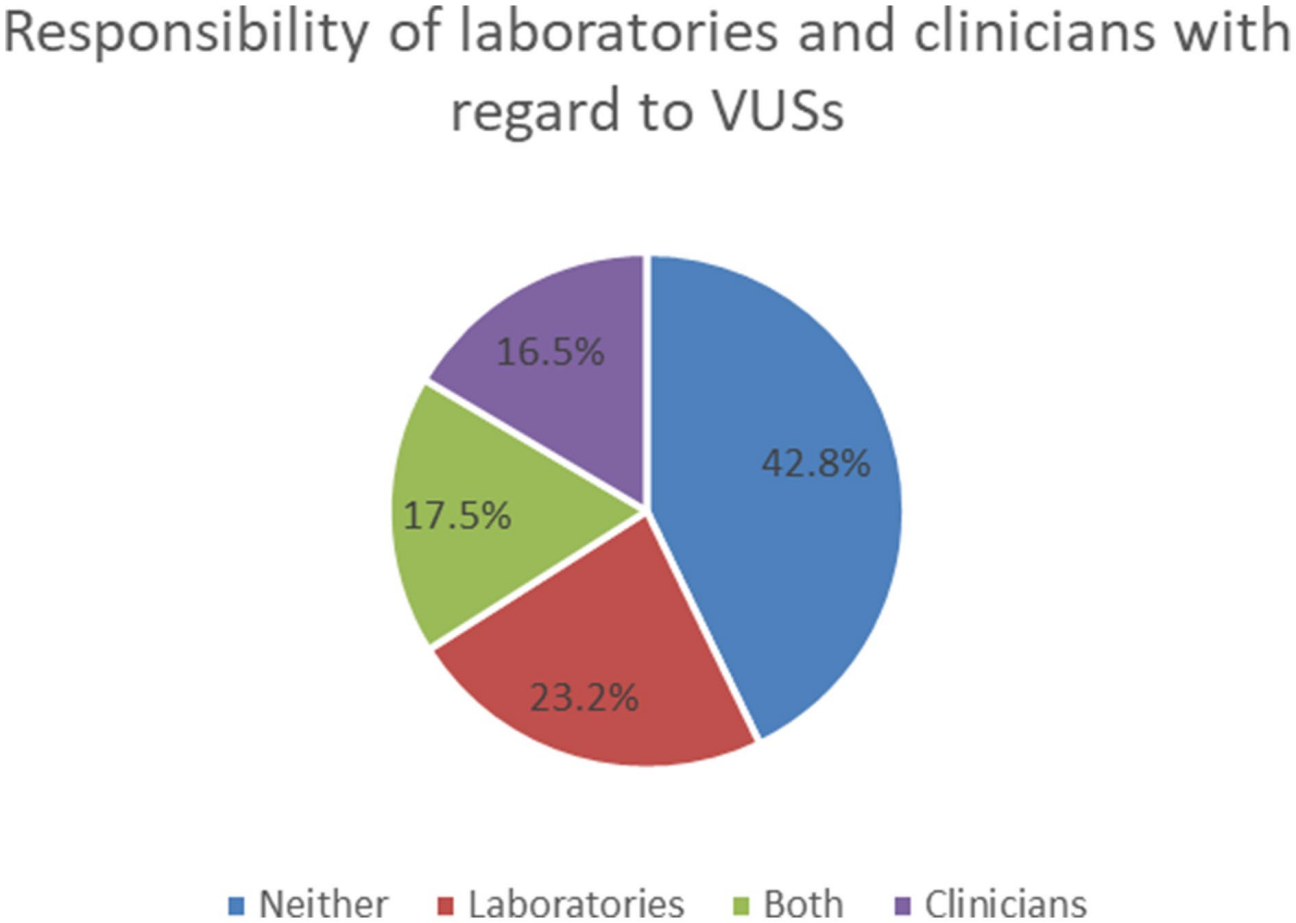
Responsibility of laboratories and clinicians with regard to VUSs.

With regard to the responsibility of laboratories and clinicians about VUSs, 42.8% of HPs felt that neither the laboratory nor the clinician was responsible for reanalyzing or recontacting the patients when VUSs were reclassified many years after the original test, 23.2% thought it was the responsibility of laboratories, 16.5% thought it was the responsibility of clinicians, 17.5% considered a joint responsibility for both (Figure 3).

## 4 Discussion

### 4.1 Main findings

#### 4.1.1 Most parents did not have the option of opting out of reporting UR from pES in pre-test counseling

Several studies looking at parental attitudes of UR in the pES were performed in the last decade (16, 17). Parents had different thresholds when receiving UR. Some parents wanted to know as much information as possible despite the potential for receiving UR, while other parents did not want to receive such information (28–31). In our study, most HPs reported that in their practice, parents were not given the option to opt out of receiving UR from pES.

However, UR from pES may bring some negative effects.

Firstly, UR could affect decision-making and clinical management. Five studies showed that parents found making decisions based on UR challenging, in particular whether to continue or terminate the pregnancy (12, 13, 18, 32, 33).

Secondly, UR can have adverse psychological effects on individuals, causing fear, diminished sense of well-being, and avoidance of decision-making (14, 15). Participants from six studies reported feeling shocked and worried on receiving UR (12, 13, 17, 30, 32, 33). Participants in some studies depicted wishing that they did not receive the UR (12, 13, 32). Five studies reported that participants felt overwhelmed by the future and lack of control over the uncertain situation following UR (13, 29, 30, 32, 33).

Thus, the fact that most HPs in this study did not work in a setting where opting out of UR was an option means that most parents may suffer from the negative impact of UR.

In addition, UR could also have a negative impact on the doctor-patient relationship, as parents sometimes react angrily when they are struggling to make decisions about the pregnancy (21). Our study investigated whether reporting UR affected the doctor-patient relationship. Most people chose “depends on the parents’ attitude towards UR,” and only 8.4% thought it would not affect the doctor-patient relationship. This result indicates that the parents’ attitude toward UR determines whether UR affects the doctor-patient relationship. We should assess parents’ tolerance for UR in pre-test counseling and give parents the option of non-disclosure of UR.

#### 4.1.2 More than half of the HPs in our study believed that UR should be reported

Several studies have been published over recent years looking at HPs’ attitudes and practice toward receiving UR from pES (21, 34–37). These findings emphasized differences in views and practices on UR across countries (23). There are few studies on HPs’ attitudes toward UR from pES in China. This study is specific to practice in China. In our study, more than half of the HPs believed that UR should be reported. Buchanan et al. has shown that women from China consider reporting of VUS and SFs as significantly more important than parents from other countries (38). The views of HPs reported in our study might reflect what parents in China want.

However, are HPs well prepared to deal with the possible negative impact of reporting UR? Once an uncertain result reported, the question then arises of how this information is being understood and handled by patients. In addition, HPs’ knowledge of UR or skills concerning communicating results also impact parents’ experience and ability to cope with uncertainty (19). Genetic information is particularly difficult for patients to understand, let alone these UR, which can complicate decision-making, such as termination of pregnancy. Meanwhile, UR may also cause psychological distress (14, 15).

Firstly, should UR affect pregnancy decisions made by the clinical team? The ACMG’s guidelines for the interpretation of sequence variants address the issue of management as follows: A variant of uncertain significance should not be used in clinical decision making (39). Our study showed that most HPs discussed with the parents but did not make any recommendations for managing the pregnancy after reporting UR, suggesting that UR did not influence the pregnancy recommendation made by most HPs. However, UR may affect the pregnancy decisions made by parents (40). Some study showed that uncertainty about the prognosis of the baby and the general lack of information about the future made it difficult for parents to make decisions (12).

Secondly, UR may also cause psychological distress, such as anxiety, overwhelmed. When parents felt overwhelmed by the UR, our study showed that about half of the respondents requested a senior member of staff, and a minority found themselves in a dilemma or made no recommendation. This result implied that the majority of respondents did not identify as having the level of seniority that would warrant them being able to manage parents who are overwhelmed without assistance from someone more experienced. In some studies, parents have expressed that how UR are communicated significantly impacts their experience and ability to cope with uncertainty (19). Those providing pre-test and post-test counseling should also have the training to support parents in managing uncertainty and making decisions about their pregnancy (41). Some attention has been given to how HPs can help manage parental uncertainty in the prenatal settings (41–43), such as establishing conceptual models of uncertainty and uncertainty management strategies.

#### 4.1.3 Responsibility of laboratories and clinicians with regard to VUSs

One unique characteristic of VUSs, compared to other types of ambiguous medical test results, is that while the result itself may remain static, its meaning is often resolved over time, as more data are gathered (44). When more evidence becomes available, variants can be reclassified. They may be “upgraded” to pathogenic or “downgraded” to benign (44). When this occurs, it raises questions about the responsibility of laboratories and clinicians with regard to VUSs.

Previous investigation with clinicians, scientists, genetic counselors and patient representatives found that patient representatives supported the reinterpretation of results over time more than other participant groups (21). Our study investigated the HPs’ views of the reanalysis and recontacting the patients when VUSs were reclassified many years after the original test. We also investigated their views on the responsibility of laboratories and clinicians with regard to VUSs (Figure 3).

In our survey, only 21.4% of HPs would recontact the patients. This question is less troublesome in the case of a downgraded VUS result, but the importance of an upgraded result making its way to a patient could be quite significant (44). Regarding responsibility for recontacting patients, we can refer to the ACMG statement (45) and recommendations of the European Society of Human Genetics (ESHG) (46). However, the responsibility for reinterpreting genetic data remains controversial (47, 48). There is a need for more evidence, including economic and utility of information for people, to inform which strategies provide the most cost-effective use of healthcare resources for recontacting (46).

### 4.2 Strengths and limitations

The strengths of the study include that our survey was completed by a wide range of HPs working in the prenatal setting. This included HPs of different specialties, years of practice, education levels, and practice setting. Furthermore, the survey was distributed nationally, giving a wide geographic distribution of thoughts and beliefs (Supplementary Figure S2).

The first limitation of the study is the limited sample size. The questionnaire should be validated in a larger sample to obtain additional information. Second, we should also acknowledge that the national survey population is not evenly distributed geographically, which affects the sample’s representativeness. Although our survey was a random questionnaire conducted nationwide, the different enthusiasm of participants in different provinces led to the uneven distribution of the number of participants in different provinces. The distribution was heavily weighted to the south and north-east of the country with fewer responses across the centre. Further research should add data in the provinces with small participants. Third, respondents were also unevenly distributed by gender. More than 90% of the participants were female, which may have affected the sample’s representativeness. Further research should focus on recruiting more male participants.

## 5 Conclusion

Most parents did not have the option to opt out of reporting UR from pES in pre-test counseling. UR did not influence the pregnancy recommendation made by most HPs. UR from pES should be discussed with parents as part of the pre-test informed consent discussion. Establishing national guidelines for reporting UR from pES and developing strategies to improve counseling skills may help HPs manage UR.

## Data availability statement

The raw data supporting the conclusions of this article will be made available by the authors, without undue reservation.

## Ethics statement

Approval was granted by the Ethics Committees of Affiliated Xiaoshan Hospital, Hangzhou Normal University (25th January, 2022/no. KL2022011). All participants gave their informed consent in this study.

## Author contributions

DL: Conceptualization, Writing – original draft. JY: Conceptualization, Formal analysis, Supervision, Writing – original draft, Writing – review & editing. WS: Data curation, Resources, Writing – review & editing. MC: Conceptualization, Data curation, Supervision, Writing – review & editing.

## Footnotes

^1^ https://www.wjx.cn/

## Supplementary material

SUPPLEMENTARY FIGURE S1 Questionnaire.

SUPPLEMENTARY FIGURE S2 The geographical distribution of respondents.
