# Supplementary material for: Attitudes toward uncertain results from prenatal exome sequencing: a national survey among healthcare professionals working in the prenatal setting
Source: Front Med (Lausanne). 2024 May 15;11:1335649. doi: 10.3389/fmed.2024.1335649 (PMC11133618; doi:10.3389/fmed.2024.1335649)
Supplement: SUPPLEMENTARY FIGURE S1 — Questionnaire. [file Table_1.DOCX]

**Uncertain Results from prenatal exome sequencing**

（The questionnaire was in Chinese. This is a translated version）

Dear Participants,

You will participate in a questionnaire entitled "Uncertain results from prenatal exome sequencing." Before participating in this survey, please read the informed consent carefully and decide whether you want to participate or not.

(1) Objective: To investigate the attitude of healthcare professionals (HPs) towards uncertain results from prenatal exome sequencing. Uncertain results from prenatal exome sequencing in our study include variants of uncertain significance (VUS), variants with variable penetrance/expressivity (VVPE), and secondary findings unrelated to the indication for testing (SFs).

(2) Methods: Collect questionnaire data from respondents, conduct statistical analysis, and draw conclusions.

(3) This survey is anonymous, and the personal information is strictly confidential.

(4) The survey is free of charge to you. All costs will be paid by the investigator.

(5) Participation in the survey is completely voluntary. You may withdraw from the survey at any time for any reason, and no information will be retained after you have withdrawn.

The researchers promise that there is no commercial interest in this survey and that your information will be kept strictly confidential.

We thank you for your support. Participating in this survey means that you recognize our commitment and are willing to support our research.

1. Do you agree to participate in this survey? [single choice] *

| ○Yes, I agree. | ○No, I don't agree. (Skip to the end of the questionnaire and submit) |
| --- | --- |

2. Have you had any experience with prenatal exome sequencing? [single choice] *

| ○Yes |
| --- |
| ○No |

**Section 1: Demographic data**

3. What is your specialty？ [single choice] *

| ○Specialist in prenatal diagnosis and fetal medicine |
| --- |
| ○Obstetricians |
| ○Technicians in prenatal diagnosis laboratories |
| ○Clinical geneticists |
| ○Laboratory geneticists |
| ○Genetic counselors |

4. How many years have you worked in your current practice? [single choice] *

| ○0-5 |
| --- |
| ○6-10 |
| ○11-20 |
| ○>20 |

5. What is the level of your experience? [single choice] *

| ○Junior |
| --- |
| ○Intermediate |
| ○Senior |

6. How old are you? (years) [gap filling] *

_________________________________

7. What is your gender? [single choice] *

| ○Male | ○Female |
| --- | --- |

8. Which ethnic group do you belong to? [single choice] *

| ○Han | ○Ethnic minorities |
| --- | --- |

9. What religion are you? [single choice] *

| ○None | ○Christian |
| --- | --- |
| ○Buddhism | ○Muslim |
| ○Others |  |

10. What is your education level? [single choice] *

| ○Doctor | ○Master |
| --- | --- |
| ○Bachelor | ○College degree and below |

11. Which of the following best describes your practice setting? Please select one. [single choice] *

| ○General hospital |
| --- |
| ○Maternal and Neonatal hospital |
| ○Genetic hospital |
| ○Private practitioner |
| ○Urban or rural primary healthcare center |
| ○Genomics Institution |
| ○Other, please specify: _________________ |

**Section 2: Practice**

12. Who decides VUS to report in your practice setting? [single choice] *

| ○Specialists in prenatal diagnosis and fetal medicine |
| --- |
| ○Technicians in prenatal diagnosis laboratories |
| ○Clinical geneticists |
| ○Obstetricians |
| ○Laboratory geneticists |
| ○MDT |
| ○Gave patients the option of opting out of reporting VUS |

13. Who decides VVPE to report in your practice setting? [single choice] *

| ○Specialists in prenatal diagnosis and fetal medicine |
| --- |
| ○Technicians in prenatal diagnosis laboratories |
| ○Clinical geneticists |
| ○Obstetricians |
| ○Laboratory geneticists |
| ○MDT |
| ○Gave patients the option of opting out of reporting VVPE |

14. Who decides SFs to report in your practice setting? [single choice] *

| ○Specialists in prenatal diagnosis and fetal medicine |
| --- |
| ○Technicians in prenatal diagnosis laboratories |
| ○Clinical geneticists |
| ○Obstetricians |
| ○Laboratory geneticists |
| ○MDT |
| ○Gave patients the option of opting out of reporting SFs |

15. Who provides pre-test counseling in your practice setting? [single choice] *

| ○Specialists in prenatal diagnosis and fetal medicine |
| --- |
| ○Obstetricians |
| ○Clinical geneticists |
| ○Laboratory geneticists |
| ○MDT |

16. Who provides post-test counseling in your practice setting? [single choice] *

| ○Specialists in prenatal diagnosis and fetal medicine |
| --- |
| ○Obstetricians |
| ○Clinical geneticists |
| ○Laboratory geneticists |
| ○MDT |

**Section 3: Attitudes**

17. What is your opinion on the return of VUS from prenatal exome sequencing? [single choice] *

| ○VUS should be returned to pregnant women |
| --- |
| ○VUS should not be returned to pregnant women |
| ○Pregnant women's autonomy should be respected in pre-test counseling |

18. What is your opinion on the return of VVPE from prenatal exome sequencing? [single choice] *

| ○VVPE should be returned to pregnant women |
| --- |
| ○VVPE should not be returned to pregnant women |
| ○Pregnant women's autonomy should be respected in pre-test counseling |

19. What is your opinion on the return of SFs from prenatal exome sequencing? [single choice] *

| ○SFs should be returned to pregnant women |
| --- |
| ○SFs should not be returned to pregnant women |
| ○Pregnant women's autonomy should be respected in pre-test counseling |

20. Whether uncertain results from prenatal exome sequencing affect the doctor-patient relationship when parents were not aware of uncertain results before testing? [single choice] *

| ○Yes |
| --- |
| ○No |
| ○Depends on the parents' attitude towards the uncertain results |

**Section 4: Recommendations after reporting UR**

21. What is your recommendation after reporting VUS? [single choice] *

| ○Continuing the pregnancy |
| --- |
| ○Terminating the pregnancy |
| ○Discussed with the parents but didn't make any recommendations |

22. What is your recommendation after reporting VVPE? [single choice] *

| ○Continuing the pregnancy |
| --- |
| ○Terminating the pregnancy |
| ○Discussed with the parents but didn't make any recommendations |

23. What is your recommendation after reporting SFs? [single choice] *

| ○Continuing the pregnancy |
| --- |
| ○Terminating the pregnancy |
| ○Discussed with the parents but didn't make any recommendations |

24. What is your recommendation to the parents who felt overwhelmed by the uncertain results from prenatal exome sequencing? [single choice] *

| ○Provided my own option of termination or continuation of the pregnancy |
| --- |
| ○No recommendation |
| ○I found myself in a dilemma and could do nothing to help parents |
| ○Signposted parents to psychological support |
| ○Referral |
| ○Requested consultation by senior staff |

**Section 5:** **Views of the reclassification of the VUS**

25. What is your view of the reanalysis and recontacting the patients when VUSs were reclassified many years after the original test? [single choice] *

| ○I wouldn't reanalyse the results |
| --- |
| ○I would reanalyse the results and recontact the patients |
| ○I would reanalyse the results but wouldn't recontact the patients |

26. Who is responsible for reanalyzing or recontacting the patients when VUSs are reclassified many years after the original test? [single choice] *

| ○Laboratories |
| --- |
| ○Clinicians |
| ○A joint responsibility for both laboratories and clinicians |
| ○Neither the laboratories nor the clinicians |
