# Supplementary material for: Attitudes toward uncertain results from prenatal exome sequencing: a national survey among healthcare professionals working in the prenatal setting
Source: Front Med (Lausanne). 2024 May 15;11:1335649. doi: 10.3389/fmed.2024.1335649 (PMC11133618; doi:10.3389/fmed.2024.1335649)

The geographical distribution of respondents


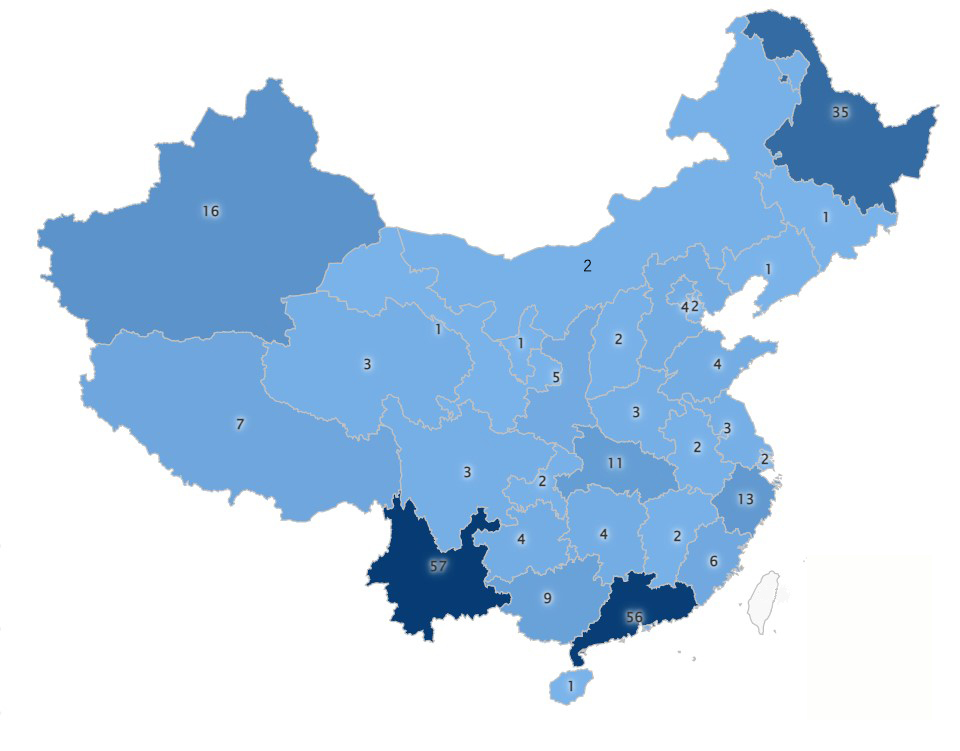


The figures on the map denote the number of surveys received in that region


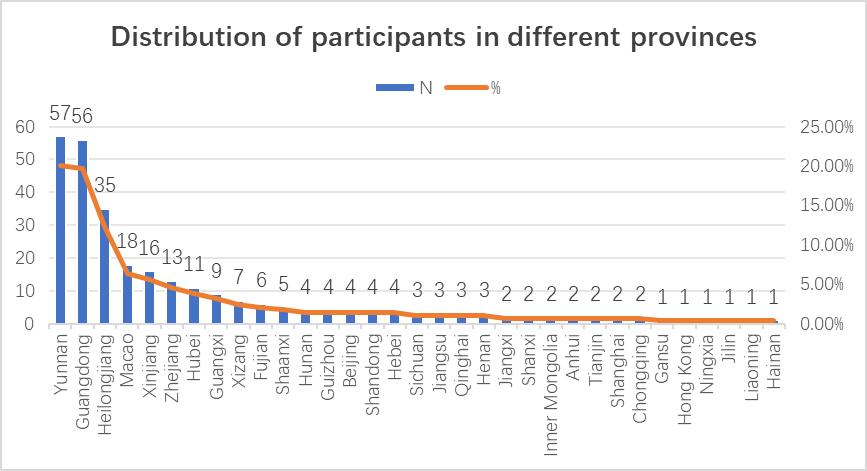

Supplement: SUPPLEMENTARY FIGURE S2 — The geographical distribution of respondents. [file Data_Sheet_1.docx]
